# Using cluster analysis to explore COVID-19 vaccine booster hesitancy by levels of medical mistrust in fully vaccinated US adults

**DOI:** 10.1080/07853890.2024.2401122

**Published:** 2024-09-11

**Authors:** Kirsten Paulus, Sarah Bauerle Bass, Whitney Cabey, Katie Singley, Caseem Luck, Ariel Hoadley, Molly Kerstetter, Alexandru-Mircea Rotaru, Elizabeth Knight, Swathi Murali, Shreya Verma, Imani Wilson-Shabazz, Heather Gardiner

**Affiliations:** a Department of Social and Behavioral Sciences, Temple University College of Public Health, Philadelphia, Pennsylvania, USA; b Risk Communication Laboratory, Temple University College of Public Health, Philadelphia, Pennsylvania, USA; c Lewis Katz School of Medicine, Temple University, Philadelphia, Pennsylvania, USA

**Keywords:** COVID-19, vaccine hesitancy, booster, infectious disease, medical mistrust

## Abstract

**Background:**

Underlying causes of vaccine hesitancy could significantly affect successful uptake of the SARS-CoV2 vaccine booster doses during new waves of COVID-19. Booster rates among US adults are far below what is needed for immunity, but little is known about booster hesitancy among fully vaccinated adults and whether medical mistrust exacerbates barriers to uptake.

**Methods:**

A cross-sectional survey was completed among 119 adults in Philadelphia, PA who reported having received the primary SARS-CoV2 vaccine series but not a booster dose. Using the LaVeist Medical Mistrust (MM) Index, a k-means cluster analysis showed two clusters (Low MM, High MM) and differences in attitudes and perceptions about COVID-19 booster vaccines were assessed using F-tests.

**Results:**

Respondents were 62% Black and female; mean age was 41; 46% reported earning less than $25,000 and 53% had a high school education or less. Overall intention to get boosted was low (mean 3.3 on 0-10 scale). Differences in COVID-19 booster perceptions between those with High (*n* = 56) vs. Low (*n* = 59) MM were found, independent of any demographic differences. Most statements (7/10) related to reasons to not be boosted were significant, with those with High MM indicating more concern about feeling sick from the vaccine (F=-3.91, *p*≤ .001), beliefs that boosters are ineffective for vaccinated people (F= −3.46, *p*≤ .001), and long-term side effect worries (F=-4.34, *p*≤ .001). Those with High MM were also more concerned about the adverse effects of the vaccine (F=-2.48, *p*=.02), but were more likely to trust getting information from doctors or healthcare providers (F= −2.25, *p*=.03).

**Conclusions:**

Results indicate that medical mistrust is an important independent construct when understanding current COVID-19 booster hesitancy. While much work has looked at demographic differences to explain vaccine hesitancy, these results suggest that further research into understanding and addressing medical mistrust could be important for implementing interventions to increase booster rates.

## Introduction

Vaccine hesitancy has become an increasing threat to public health, most recently manifested during the COVID-19 pandemic. In the US, vaccination rates indicated certain groups were less likely to be vaccinated, including Black/African Americans, younger adults, white men with less than a college education, and those with a conservative ideology [1–5]. While the US had a close to 70% overall vaccination rate (completing the primary series), the emergence of COVID-19 variants has threatened the progress made with vaccinations [6]. For example, the Omicron variant resulted in record-high COVID-19 case counts regardless of vaccination status [6]. However, booster shot doses in the fully vaccinated population can bolster waning immunity and protect vaccinated individuals from COVID-19 variants. This would be helpful for SARS-CoV-2 variants such as Omicron or the new subvariant of Omicron, JN.1, which now makes up over 60% of new US COVID cases [7]. Since January 2022, the US Centers for Disease Control and Prevention (CDC) recommended an mRNA booster dose after receiving the primary vaccination series, which has been shown to decrease transmission, as well as hospitalization and emergency room visits [8,9]. However, only 17% of the total US population 5 years of age or older has received a booster [6]. In Philadelphia, where this research occurred, there are even lower rates of booster uptake among Black and Hispanic residents [10,11]. The divide between those who are booster eligible—those who have already received the primary vaccination series—and those who are boosted must be narrowed to adequately protect the public from future waves of the SARS-CoV-2 virus.

Understanding the drivers of booster hesitancy can help public health professionals intervene to encourage COVID-19 booster uptake. But getting people to accept vaccination is a complex decision, especially a vaccine that has been the subject of significant mis/disinformation [6,10–14]. While work has been done to understand barriers to uptake of the primary COVID-19 vaccine, it is unclear what is driving low rates of booster uptake in the US, since those eligible have already made the decision to get the primary vaccine series. Studies that have looked at COVID-19 booster acceptance and/or hesitancy have been done among non-US populations using web-based surveys, which limits the generalizability to the US adult population [11–16]. The few that have looked at US adults have shown that just over 20% of fully vaccinated U.S. adults are unsure or hesitant to get a recommended booster dose [17,18]. Concerns include negative experiences with side effects from prior COVID-19 vaccine doses, uncertainties about booster side effects, worry about chronic health issues or allergic reactions, and no mention of need for boosters when getting the primary vaccine series [19].

One topic that has yet to be explored as a potential barrier to COVID-19 booster uptake is medical mistrust. Defined as the lack of trust or suspicion of any medical organizations, including health providers and treatment themselves [20,21], medical mistrust is rooted in the historical and current mistreatment of those socially and economically marginalized populations. It has been found to be an important construct connected to health behaviour, medical decision making and patient-provider communication [20,21]. Mistrust of research, pharmaceutical companies and governments in general has also specifically been associated with the SARS-CoV-2 pandemic. In the US, it has been connected to the distrust of scientific information about transmission, need for protective measures like mask wearing and social distancing, and the vaccine, where many people believed it was simply another form of experimentation [20,21]. Mistrust also affected initial vaccine uptake [21] and facilitated the relationship between conspiracy beliefs and vaccine hesitancy [21].

Inherently, medical mistrust is often connected to race/ethnicity, particularly through evidence of experimentation on racial and ethnic minorities such as the Tuskegee Syphilis study between 1932 and 1972 [20]. This historical evidence, along with documented lower levels of health literacy [22]; sociocultural factors such as media representation, racial discrimination and community influences [22]; and less or inadequate health insurance coverage [22], as well as specific attitudes or fears about how they might be treated in the healthcare system, all contribute to a distrust by racial and ethnic minorities [22]. These feelings thus extend beyond the history of the Tuskegee experiment [22], reinforced by contemporary discriminatory practices in the healthcare system [22] that affect individual level participation in healthcare decision making, but also trust of system level communication, such as widely disseminated information and messages about COVID-19 booster vaccinations [23,24].

Importantly, there is evidence that medical mistrust has been a barrier to COVID-19 vaccination uptake especially among minoritized populations, including whether someone received more than one [25]. Bogart et al. found that 97% of Black or African American participants expressed mistrust of the COVID-19 vaccine [26]. This is not surprising, as Black Americans have shown to be significantly more likely to report ‘no’ or ‘not sure’ to questions about willingness to uptake a vaccine when compared to White counterparts. For COVID-19 specifically, many Black individuals feared that the rapid development and promotion of the vaccine in their communities, especially in a sociopolitical climate they perceived to be hostile, was evidence to distrust the vaccine and other communication about COVID-19 mitigation efforts [27]. Studies in 2021 and 2023 found that combined perceived discrimination experienced by Black Americans every day fuels medical mistrust and thus results in a lower odds of having the COVID-19 vaccine compared to those that do not experience discrimination [27,28]. When the level of suspicion of the COVID-19 vaccine is high, evidence suggests this outweighs any other potential facilitator, such as it being a social responsibility or a way to keep oneself from being sick [29]. Whether that holds true for COVID-19 booster shots is unknown.

Thus, medical mistrust may be an important construct in understanding why previously vaccinated adults have not made the decision to have a COVID-19 booster shot, especially as COVID-19 resurges in communities [27–30]. But its impact on barriers and facilitators is poorly understood. This study aims to understand whether medical mistrust and its association with barriers and facilitators could help explain the decision-making process to receive a COVID-19 booster. The main research question is whether those with more medical mistrust will report more barriers and fewer facilitators to receiving a COVID-19 booster compared to those with lower levels of medical mistrust. This exploratory research could be used to inform future interventions that aim to address medical mistrust and encourage proper prevention strategies—such as booster uptake—in a future disease outbreak in the United States.

## Methods

These analyses utilized data from a quantitative survey that was part of a mixed-methods study to explore experiences with COVID-19 vaccination, understand drivers of intent to receive a booster dose and elucidate psychosocial, experiential and structural barriers to receipt of a COVID-19 booster dose. The purpose of this formative work was to provide data to inform the development of a community-based COVID-19 booster communication campaign. The cross-sectional survey was conducted among residents of Philadelphia, PA who reported receiving their primary vaccinations but not their booster shots. Data were collected from March through May 2023.

Participants were recruited a number of ways through convenience sampling strategies, including in person at community organizations across the city—at a major safety-net hospital—at planned events or locations and online. At in-person events, staff asked people if they would be interested in participating and if interested, screened to determine eligibility. Participants could take the survey either on an iPad or their own device or on paper as self-administered or read to them by study staff. Online recruitment was completed by asking community organizations to post information to their social media accounts, as well as through posted flyers or recruitment cards that were available at various city locations. All materials had a QR code, URL and phone number. Participants could scan or click on the link, which would take them to a secure eligibility and survey link, or, if they preferred, could call study staff and complete the survey over the phone. Surveys utilized Qualtrics, the secure online survey software [31]. All participants provided written informed consent and if completed online, IP addresses were checked to ensure it was located in Philadelphia. Data collection occurred between December 2022 and May 2023. All participants received a $15 gift card upon completion. Temple University’s Institutional Review Board reviewed and approved this study (#29430).

### Participants

Eligible participants (*n* = 119) were: (1) 18 years of age or older; (2) Able to speak and read English; (3) Reported living in Philadelphia County; (4) Self-reported that they are fully vaccinated against COVID-19; and (5) Had not received a COVID-19 booster shot.

### Measures

The survey instrument developed by the authors used both validated measures as well as study-specific items based on findings from qualitative interviews [32] and previous research on COVID-19 vaccine hesitancy [33]. It consisted of sociodemographic questions and sections related to COVID-19 experience and knowledge, vaccine acceptance and literacy, information sources and trust, willingness to get the booster, beliefs about the booster, reasons not to get the booster and barriers to getting the booster. All sections were presented in blocks of statements for respondents to agree or disagree with on a zero to 10 scale (zero = highly disagree, 10 = highly agree).

The following validated scales were used;

- **Vaccine Acceptance**: Yadete et al.’s nine item vaccine acceptance scale assesses the overall knowledge about COVID-19 vaccines [17]. These included that vaccines are important for health, they are effective, they protect from diseases and the people should follow recommendations of doctors about vaccination.
- **Vaccine Literacy:** Biasio et al.’s nine item assessment of COVID-19 vaccine literacy was adapted for the COVID-19 booster [34]. Items assess whether the respondent consulted more than one source of information, if they understood the information and if they discussed what they had found out about the COVID-19 booster with their doctor or other people.
- **Conspiratorial Thinking:** Bruder et al.’s five item Conspiracy Mentality Questionnaire was used to measure conspiratorial thinking [35]. These items measure how much a respondent believes in conspiracies (e.g. ‘Important things happen in the world the public is never informed about’).
- **Medical Mistrust:** LaVeist et al.’s seven item Medical Mistrust Index was used to measure levels of mistrust [36]. Items assess how much a respondents believes that institutions may or may not be trustworthy (e.g. ‘Healthcare organizations have sometimes done harmful experiments on patients without their knowledge’).

Other survey items were informed by the Centers for Disease Control and Prevention survey items on barriers to getting the COVID-19 vaccine [34], the Kaiser Family Foundation COVID-19 Vaccine Monitor survey from July 2022 [28], as well as the study team’s previous work on COVID-19 vaccine hesitancy [33] and formative qualitative interviews with those who had been vaccinated but not boosted [32]. All sections were presented in blocks of statements for respondents to agree or disagree with on a zero to 10 scale (zero = highly disagree, 10 = highly age). These sections included:

- **Beliefs about COVID-19:** 6 items, related to conspiratorial beliefs about COVID-19 (e.g. ‘COVID-19 is a hoax’)
- **Beliefs about COVID-19 Boosters**: Items (n = 9) assessed beliefs about the COVID-19 boosters (e.g. ‘I believe the COVID-19 vaccine booster shots are being pushed just to make more money’ and ‘I believe getting the COVID-19 vaccine booster shots help protect me and my family/friends’).
- **Reasons to be boosted:** Items (n = 6) assessed the perceived reasons someone might want to get boosted. (e.g. ‘Getting the COVID-19 booster is the only way to stay safe from getting COVID-19 in the future’).
- **Reasons to not be boosted:** Items (n = 10) assessed the perceived reasons to not get boosted (e.g. ‘I had COVID-19 and didn’t get very sick so I don’t think a booster is necessary’).
- **Barriers to getting boosted:** Items (n = 10) assessed potential structural or personal barriers to getting boosted (e.g. ‘I have a medical reason that makes me ineligible to get boosted’, and ‘The place to get a booster shot is too far away’).
- **Trust of Information Sources:** Items (n = 12) assessed what information sources people were the most trusting of and who they got their information about COVID-19 from the most often. These included friends or family, doctors, pharmacists, official government websites, the CDC, the city Department of Public Health, politicians, news networks, radio and social media.

Finally, one item measured participants’ self-described willingness to get the COVID-19 booster, in line with the Diffusion of Innovation Theory’s Adopter categories [37]. Since none of the respondents had yet been boosted, categories included: Early Majority (‘I plan to get boosted but just haven’t gotten around to it’), Late Majority (‘I will get the booster shot if people I trust tell me that I should get it’) and Laggards (‘I’d rather wait to get the booster shot until everyone else has gotten it’). A ‘refuser’category was also added because of preliminary work that indicated a number of those would not get the booster under any circumstances [32], despite having already been vaccinated. This was operationalized with two statements (‘I don’t want to get the booster shot because I’m not really sure I need it’ and ‘I will never get the booster shot’).

### Analytic plan

To examine associations between our constructs of interest, we performed a k-means cluster analysis using items from the LaVeist Medical Mistrust Index (MM) scale [36]. A non-hierarchical method is used in the k-means approach to clustering to discern latent subgroups within a sample. Individual cases are then assigned to a predetermined number of clusters according to their proximity to the nearest centroid (mean) of constituent items [38]. This is then performed iteratively until the desired number of clusters is produced. Four cases were excluded because of missing data, reducing the total sample size from 119 participants to *n* = 115. From this, a two-cluster solution was found (Low MM, *n* = 59 and High MM, *n* = 56). Once a cluster solution was found, the associations between membership in one of the two clusters and survey items were assessed using F-tests to test for potential differences in perceptions of and attitudes about COVID-19 booster vaccines, including perceived booster barriers and facilitators. An alpha value of 0.05 was used to determine statistical significance. Chi square tests were used to compare demographics between clusters. All analyses were done with SPSS v. 28.

## Results

Differences between clusters based on their constituent items were analyzed to create definitions for both clusters. Table 1 reports the means for each item delineated by cluster. We use the terms Low Medical Mistrust (MM) for Cluster 1 and High Medical Mistrust (MM) for Cluster 2 to apply common nomenclature to denote how segments differed by the study variables.

**Table 1.** Differences between two groups on clustering variables.

	Cluster 1 (*n* = 59) Low medical mistrust (MM) Means	Cluster 2 (*n* = 56) High medical mistrust (MM) Means	*p*
You’d better be cautious when dealing with health care organizations.^a^	4.00	7.00	<0.001
Patients have sometimes been deceived or misled by health care organizations.^a^	4.00	7.00	<0.001
When health care organizations make mistakes they usually cover it up.^a^	4.00	8.00	<0.001
Health care organizations have sometimes done harmful experiments on patients without their knowledge.^a^	3.00	8.00	<0.001
Health care organizations don’t always keep your information totally private.^a^	4.00	7.00	<0.001
Sometimes I wonder if health care organizations really know what they are doing.^a^	4.00	7.00	<0.001
Mistakes are common in health care organizations.^a^	4.00	8.00	<0.001

^a^The range of 0-10, 0 = completely disagree to 10 = completely agree.

### Sample demographics

Overall, a majority of the sample was African American/Black (62.1%), finished high school or below (53.4%), had health insurance (92.1%), identified as Democrat (53.5%) and were female (61.9%). Almost half made less than $24,999 a year in income (46.1%). Average age was 41.43 years. Table 2 presents a summary of demographics by total sample and by cluster along with other variables of interest. No significant differences were observed between clusters on any demographic variables.

**Table 2.** Demographics.

Item	Full sample (*n* = 119)	Cluster 1 (*n* = 59) Low medical mistrust	Cluster 2 (*n* = 56) High medical mistrust	*p*
**Insurance (yes)**	92.1%	88.1%	98.2%	0.09
**Age (mean)**	41.43	39.66	43.02	0.19
**Race**				0.87
African American/Black	62.1%	59.3%	63.6%	
Caucasian/White	16.4%	16.9%	16.4%	
Other	21.6%	23.7%	20.0%	
**Ethnicity**				0.17
Not Hispanic	72.6%	66.7%	78.2%	
Hispanic	27.4%	33.3%	21.8%	
**Gender**				0.20
Male	37.3%	42.4%	28.6%	
Female	61.9%	57.6%	69.6%	
Other	0.9%	0.0%	1.8%	
**Education**				0.19
High school or below	53.4%	47.5%	57.1%	
Some college or full degree	39.0%	47.5%	32.1%	
Above college degree	7.7%	5.1%	10.7%	
**Income**				0.440
Less than $24,999	46.1%	44.8%	45.5%	
$25,000 to $54,999	26.1%	31.0%	21.8%	
$55,000+	27.8%	24.1%	32.7%	
**Political Affiliation**				0.39
Democrat	53.5%	49.2%	57.1%	
Non-Democrat	46.5%	50.8%	42.9%	

### Perceptual variables

Chi square *p*-values and group distributions for Diffusion of Innovation adopter categories are reported in Table 3. Means, standard deviations, F-values, *p*-values and Cohen’s d are reported in Table 4 for all survey items in survey sections where there was at least one significant difference by cluster. Significant results are described below (at the 0.05 significance level or below).

**Table 3.** Differences between clusters on diffusion of innovation.

Item	Cluster 1 (*n* = 59) Low medical mistrust	Cluster 2 (*n* = 56) High medical mistrust	*p*
**Diffusion of Innovation**			0.03*
I plan to get the booster shot but just haven’t gotten around to it.	28.8%	17.9%	
I will get the booster shot if people I trust tell me that I should get it.	6.8%	12.5%	
I’d rather wait to get the booster shot until everyone else has gotten it.	22.0%	15.7%	
I don’t want to get the booster shot because I’m not really sure I need it.	32.2%	32.1%	
I will never get the booster shot.	10.2%	28.6%	

*p < 0.05

**Table 4.** Differences between clusters on other perceptual items.

Item	Cluster 1 (*n* = 59) Low medical mistrust	Cluster 2 (*n* = 56) High medical mistrust	*F*	*p*	Cohen’s *d*
**Reasons Not To Be Boosted**					
I recently had COVID-19 and don’t need to be boosted	3.50 (2.76)	4.30 (3.94)	−1.27	0.21	3.39
I had COVID-19 and didn’t get very sick so I don’t think a booster is necessary.	3.88 (2.95)	4.43 (3.76)	−0.87	0.39	3.37
I had a friend or family member who had a bad reaction when they got the booster so I don’t want to get one.	3.62 (2.79)	5.23 (3.87)	−2.56	0.01***	3.36
The original COVID-19 vaccine made me sick and I don’t want to get sick again.	3.81 (2.92)	6.22 (3.60)	−3.91	<0.001***	3.27
I don’t think the vaccines work because I got COVID-19 after I was vaccinated.	3.54 (2.77)	5.75 (3.90)	−3.46	<0.001***	3.38
I want to wait and see what happens with other people before I get a booster shot.	4.03 (2.98)	5.79 (3.70)	−2.79	0.01***	3.35
I got the original COVID-19 vaccine so I don’t need a booster.	4.22 (2.73)	5.75 (3.69)	−2.51	0.01***	3.24
I am afraid of needles and pain and don’t want to get another shot.	3.62 (3.06)	3.48 (3.74)	0.22	0.83	3.41
I am concerned about long-term side effects of getting a booster shot.	4.60 (3.25)	7.34 (3.48)	−4.34	<0.001***	3.36
I just do not want to get a COVID-19 vaccine booster shot.	4.33 (2.85)	5.95 (3.47)	−2.73	0.01***	3.17
**Vaccine Acceptance**					
Vaccines are important for my health.	5.78 (2.88)	6.16 (3.65)	−0.62	0.54	3.27
Vaccines are effective.	5.97 (2.55)	6.45 (3.16)	−0.91	0.37	2.86
Vaccination is important for my community.	5.82 (2.80)	6.35 (3.46)	−0.88	0.38	3.14
Vaccines offered by the Government in my community are beneficial.	5.61 (2.65)	5.24 (3.40)	0.66	0.51	3.04
New vaccines are riskier than older ones.	4.39 (2.55)	5.25 (3.39)	−1.54	0.13	2.99
Vaccine information is reliable and trustworthy.	4.76 (2.59)	4.84 (2.81)	−0.15	0.88	2.70
Vaccines protect from diseases.	5.78 (2.64)	6.09 (3.38)	−0.55	0.59	3.02
I follow recommendations of doctors about vaccination.	6.15 (2.61)	6.34 (2.91)	−0.36	0.72	2.76
I’m concerned about the adverse effects of vaccines.	5.97 (2.51)	7.29 (3.17)	−2.48	0.02**	2.85
**Trust of Information Sources**					
Friends or family	4.59 (2.77)	4.55 (3.29)	0.07	0.95	3.03
Doctor or other healthcare provider	5.86 (3.21)	7.21 (3.22)	−2.25	0.03**	3.22
Pharmacist	5.51 (3.24)	6.36 (3.57)	−1.33	0.19	3.41
Official government website or information sources (state, federal, city)	5.46 (2.93)	4.69 (3.63)	−0.21	0.84	3.29
Centers for Disease Control and Prevention (CDC)	6.24 (3.12)	5.75 (3.55)	0.79	0.43	3.34
Philadelphia Department of Public Health	5.57 (3.03)	5.34 (3.55)	0.37	0.71	3.30
Politicians	3.51 (2.73)	2.78 (3.37)	1.27	0.21	3.05
National news networks	4.08 (2.96)	3.75 (3.40)	0.57	0.57	3.18
Cable news networks	3.90 (2.57)	3.78 (3.19)	0.22	0.83	2.89
Local news networks	4.69 (3.07)	3.98 (3.51)	1.16	0.25	3.29
Radio	4.10 (2.55)	3.42 (3.15)	1.27	0.21	3.86
Social media	3.56 (2.51)	3.10 (3.09)	0.87	0.39	2.80

**p < 0.01; ***p < 0.001

### Diffusion of innovation adopter categories

Distributions of adopter categories were found to be significantly different between clusters (*p* = 0.03), with the Low MM cluster more likely to say the still plan to get the booster ‘but just haven’t gotten around to it’ (i.e. ‘early majority’) (28.8% vs. 17.9%). Those with High MM were more likely to say that they would ‘never get the booster’ (i.e. ‘refuser’) (28.6% vs. 10.2%).

### Booster barriers/refusal

Seven out of ten items in this section were found to be significant. Those with high medical mistrust were more likely to agree with the following statements: ‘I had a friend or family member who had a bad reaction when they got the booster so I don’t want to get one’, (*M* = 3.62 vs. *M* = 5.23; F(113)=-2.56; *p* = 0.01); ‘The original COVID-19 vaccine made me sick and I don’t want to get sick again’, (*M* = 3.81 vs. *M* = 6.22; F(113)=-3.91; *p*= < 0.001); ‘I don’t think the vaccines work because I got COVID-19 after I was vaccinated’, (*M* = 3.54 vs. *M* = 5.75; F(113)= −3.46; *p*= <0.001); ‘I want to wait and see what happens with other people before I get a booster shot’, (*M* = 4.03 vs. *M* = 5.79; F(113)= −2.79; *p* = 0.01); ‘I got the original COVID-19 vaccine so I don’t need a booster’, (*M* = 4.22 vs. *M* = 5.75; F(113)= −2.51; *p* = 0.01); ‘I am concerned about long term side effects of getting a booster shot’, (*M* = 4.60 vs. *M* = 7.34; F(113)=-4.34; *p*= <0.001); and ‘I just do not want to get a COVID-19 vaccine booster shot’, (*M* = 4.33 vs. *M* = 5.95; F(113)= −2.73; *p* = 0.01).

### Vaccine acceptance

There was one significant item found in this section where those with High MM were more likely to say ‘I’m concerned about the adverse effects of vaccines’, (*M* = 7.29 vs. *M* = 5.97; F(113)= −2.48; *p* = 0.02).

### Information sources

Finally, there was one significant item in who the groups trusted with COVID information. Those with High MM were more trusting of doctors or other health care providers than those with Low MM (*M* = 7.21 vs. *M* = 5.86; F(113)=-2.25; *p* = 0.03).

## Discussion

The impact of medical mistrust on COVID-19 booster decision making in those who have completed their primary vaccination series has not been previously documented in US populations. The objective of this formative work was to characterize booster hesitancy in a cross-sectional convenience sample of adults living in Philadelphia who had previously been vaccinated but not boosted and assess the impact of medical mistrust on their perceptions about the barriers and facilitators to being boosted. Results identified two distinct cluster groups using the Medical Mistrust Index which did not differ by demographic characteristics, indicating it is a unique, independent variable important to understanding potential booster hesitancy in the population. This is important as other work has primarily used demographic descriptors to compare booster hesitancy across populations [17,39]. Thus, medical mistrust could be a helpful construct when thinking about developing future interventions during an ongoing pandemic [20,21,25–27].

Finding suggest that medical mistrust primarily affects perceived reasons to not get a COVID-19 booster. This suggests that aside from other social and structural barriers that Americans might face in receiving a booster, there are significant intrapersonal barriers that impede uptake, even among those who have received their primary vaccinations. Those with High MM were significantly more likely to agree with most of the reasons to not get a booster, including not thinking the vaccine works, worry about similar side effects to the primary vaccination, belief that others had had negative side effects from the booster, and wanting to wait to see ‘what happens’. This finding is important within the context of prior studies on vaccination refusal, where those who perceived stronger barriers were less likely to receive a vaccine [40–43]. This suggests that people who are less trusting of the medial system are fearful of getting the booster shot, do not believe in the booster shot as an effective way to combat COVID-19, do not trust or are concerned about the safety of the booster shot, or simply do not want to get the booster shot now that they have completed their primary vaccination series. Clearly, even though both clusters had not been boosted, mistrust of the medical system has an evident impact on perceptions of why one would or would not get boosted.

Interestingly, reasons to get the booster were not statistically different by the cluster groups. This may be driven by the amount of mis/disinformation available on COVID-19 generally, which often amplifies the vaccine’s negative side effects. Betsch et al. found that even a short exposure to content critical of any vaccine leads to increased perceived risk and decreased intention of being vaccinated [44]. Information about the COVID-19 pandemic has largely played out on social media, a first in public health. Despite most participants indicating they do not ‘trust’ social media for COVID-19 information, its ability to quickly spread mis/disinformation using first-person accounts and imagery can have an important impact on public thinking [45]. This result is similar to other findings about trust of information sources, with those saying they do trust social media being also more likely to not believe science-based sources and to attenuate their perceived risk of negative COVID-19 effects [46,47]. This, when coupled with medical mistrust, may fuel hesitancy and impact beliefs about the COVID-19 booster, counteracting any positive information about why one should be boosted [21,48,49]. However, participants with higher levels of medical mistrust also said they were more likely to trust their doctor or healthcare provider for COVID-19 booster information. This disconnect between having trust of a personal healthcare provider compared to the larger healthcare system, has been seen in other research. In fact, past research has established that the quality of patient-provider relationships can moderate the relationship between greater medical mistrust and healthcare engagement [25]. This is important in that it may provide a useful intervention strategy to get booster information to those who have more hesitancy, and has already been an established correlate to COVID-19 primary vaccination uptake [50]. This finding could be leveraged in interventions aimed at increasing booster uptake.

This study also highlights the role of Diffusion of Innovation and concern about the pandemic (and prevention of illness) in booster uptake. Those with higher levels of medical mistrust were more likely to fall in the ‘laggard’ or ‘refuser’ adopter categories, despite the finding that this group also reported reading more information about the boosters and not having significantly different levels of vaccine acceptance than those with lower medical mistrust. This further highlights the potential impact medical mistrust has on the booster decision. Since ‘laggard’ adopters are more likely to trust information from those around them (i.e. personal doctors, friends, family, religious leaders) [51], if one opinion leader is not supportive of the decision or also has higher levels of medical mistrust, positive information may be less likely to be diffused in communities. This is supported by some previous work in the context of COVID-19 vaccination [52]. Even though they may be receiving information and understand why the booster is needed, mistrust of the healthcare system by both themselves and those around them may negate this information [52,53]. As a result, future strategies to diffuse or promote the COVID-19 booster must take into consideration the already existing fear and mistrust that many communities have towards the medical system and acknowledge the importance of diffusing to whole communities, rather than just individuals. These findings suggest that using community-based approaches that use trusted messengers to talk about the COVID-19 boosters are more likely to diffuse information that is trusted. Utilizing existing community-based organizations or enlisting the help of community leaders could be an important strategy to address fears and mistrust of the medical system that may be embedded in perceived barriers and reasons to refuse the booster. These types of interventions were effective at reaching vulnerable populations with the primary vaccination series [54,55], and could serve as a model for encouraging uptake of the COVID-19 boosters.

It is clear, especially among barriers and reasons for refusal of the booster, that medical mistrust may serve as a mediator in the decision-making process of vaccine uptake. These findings can be used as a guide on how best to move individuals towards booster uptake when they have fear, mistrust and skepticism towards the medical system. However, though work by Carico et al. suggests that people will participate in preventative behaviours if they perceive it to be a large threat to their health, this is certainly not enough; the threat posed by the solution to the problem itself (the booster as a solution to COVID-19) must be addressed as well [56]. Acknowledging medical mistrust, while also promoting the booster as safe and effective, and using trusted sources of information from their community, may potentially have an effect on improving uptake of the COVID-19 booster. Since this study did not sample those who had been boosted to compare levels of mistrust across groups, future research should further explore the role of medical mistrust as a mediator for booster uptake. If it is found that medical mistrust is indeed lower in those who have been boosted, an intervention aimed at increasing trust to see if higher booster rates can be achieved would be warranted.

There are some limitations to this study. First, due to the nature of cross-sectional data from a convenience sample, temporality inferences could not be made and data may not be representative of a broader population. Additionally, the survey relies on self-reported data, so social desirability bias should be considered when evaluating results. On a larger scale, results may not be generalizable to the entire US population, as the study was conducted only in Philadelphia and among people who have received the entire primary vaccination series. Conclusions may not be able to be made for others in different geographic areas or those who have not finished their primary vaccination series. More importantly, those who are in different geographic areas may perceive the threat of COVID-19 differently, receive their information from different sources, have differing levels of vaccine literacy and have different relationships with the medical system (and thus differing levels of medical mistrust). The relatively small sample size also limits the possibility of using alternate statistical models and/or controlling for covariates. While the study sample was adequate for completing the formative analyses presented in this study, it may have resulted in less valid estimates compared to those that could be found within a larger sample size. Finally, because we only sampled those who had not been boosted, our ability to make conclusions from our findings is limited to this population.

## Conclusion

Understanding how to best balance medical mistrust and the threat from COVID-19 can better inform community-based interventions to improve future pandemic response in the US. Findings from this work indicate that better addressing medical mistrust in interventions with trusted messengers will be important to increasing the rate of booster acceptance in populations that may have higher levels of medical mistrust.

## Data Availability

The codes generated for the analysis in this current study are not publicly but are available from the corresponding author upon reasonable request.

## Data availability statement

The datasets generated and/or analyzed during the current study are not publicly available but are available from the corresponding author upon reasonable request.

## References

[1] Ndugga N, Hill L, Artiga S, et al. Latest data on COVID-19 vaccinations by race/ethnicity. KFF; 2022. [cited 2024 Jan 9]. Available from https://www.kff.org/coronavirus-covid-19/issue-brief/latest-data-on-covid-19-vaccinations-by-race-ethnicity/

[2] Reece S, CarlLee S, Scott AJ, et al. Hesitant adopters: COVID-19 vaccine hesitancy among diverse vaccinated adults in the United States. Infect Med (Beijing). 2023;2(2):89–95. doi: 10.1016/j.imj.2023.03.001. 38013742 PMC10038887

[3] Yasmin F, Najeeb H, Moeed A, et al. COVID-19 vaccine hesitancy in the United States: a systematic review. Front Public Health. 2021;9:770985. doi: 10.3389/fpubh.2021.770985. 34888288 PMC8650625

[4] Hill L, Artiga S, Ndugga N. COVID-19 cases, deaths, and vaccinations by race/ethnicity as of Winter 2022. KFF; 2023. [cited 2024 Jan 13]. Available from https://www.kff.org/coronavirus-covid-19/issue-brief/covid-19-cases-deaths-and-vaccinations-by-race-ethnicity-as-of-winter-2022/

[5] KFF. COVID-19 vaccine monitor dashboard. 2024. [cited 2024 Jan 13]. Available from https://www.kff.org/coronavirus-covid-19/dashboard/kff-covid-19-vaccine-monitor-dashboard/

[6] Centers for Disease Control and Prevention. COVID data tracker; 2020. [cited 2024 Jan 8]. Available from https://covid.cdc.gov/covid-data-tracker

[7] Centers for Disease Control and Prevention. COVID-19 activity increases as prevalence of JN.1 variant continues to rise. 2024. [cited 2024 Jan 19]. Available from https://www.cdc.gov/respiratory-viruses/whats-new/JN.1-update-2024-01-05.html

[8] Moreira ED, Kitchin N, Xu X, et al. Safety and efficacy of a third dose of BNT162b2 COVID-19 vaccine. N Engl J Med. 2022;386(20):1910–1921. doi: 10.1056/NEJMoa2200674. 35320659 PMC9006787

[9] Wald A. Booster vaccination to reduce SARS-CoV-2 transmission and infection. JAMA. 2022;327(4):327–328. doi: 10.1001/jama.2021.23726. 35006269

[10] Philadelphia Department of Public Health. Vaccine data. 2023. [cited 2024 Jan 8]. Available from https://www.phila.gov/programs/coronavirus-disease-2019-covid-19/vaccines/vaccine-data/

[11] Babicki M, Mastalerz-Migas A. Attitudes of poles towards the COVID-19 vaccine booster dose: an online survey in Poland. Vaccines (Basel). 2022;10(1):68. doi: 10.3390/vaccines10010068. 35062729 PMC8778409

[12] Abdullah NS, Ching SM, Ali H. Willingness to receive a COVID-19 booster vaccine and its associated factors among adults with chronic disease: a cross-sectional study in Putrajaya, Malaysia. Malays Fam Physician. 2023;18:30. doi: 10.51866/oa.260. 37292226 PMC10246709

[13] Folcarelli L, Miraglia Del Giudice G, Corea F, et al. Intention to receive the COVID-19 vaccine booster dose in a university community in Italy. Vaccines (Basel). 2022;10(2):146. doi: 10.3390/vaccines10020146. 35214605 PMC8877002

[14] Klugar M, Riad A, Mohanan L, et al. COVID-19 vaccine booster hesitancy (VBH) of healthcare workers in Czechia: national cross-sectional study. Vaccines (Basel). 2021;9(12):1437. doi: 10.3390/vaccines9121437. 34960183 PMC8705445

[15] Rzymski P, Poniedziałek B, Fal A. Willingness to receive the booster COVID-19 vaccine dose in Poland. Vaccines (Basel). 2021;9(11):1286. doi: 10.3390/vaccines9111286. 34835217 PMC8624071

[16] Sønderskov KM, Vistisen HT, Dinesen PT, et al. COVID-19 booster vaccine willingness. Dan Med J. 2021;69(1):A10210765. 34913428

[17] Yadete T, Batra K, Netski DM, et al. Assessing acceptability of COVID-19 vaccine booster dose among adult Americans: a cross-sectional study. Vaccines (Basel). 2021;9(12):1424. doi: 10.3390/vaccines9121424. 34960170 PMC8703732

[18] Pal S, Shekhar R, Kottewar S, et al. COVID-19 vaccine hesitancy and attitude toward booster doses among US healthcare workers. Vaccines (Basel). 2021;9(11):1358. doi: 10.3390/vaccines9111358. 34835289 PMC8617683

[19] Hahn MB, Fried RL, Cochran P, et al. Evolving perceptions of COVID-19 vaccines among remote Alaskan communities. Int J Circumpolar Health. 2022;81(1):2021684. doi: 10.1080/22423982.2021.2021684. 35057696 PMC8786257

[20] Jaiswal J, Halkitis PN. Towards a more inclusive and dynamic understanding of medical mistrust informed by science. Behav Med. 2019;45(2):79–85. doi: 10.1080/08964289.2019.1619511. 31343962 PMC7808310

[21] Zhang X, Guo Y, Zhou Q, et al. The mediating roles of medical mistrust, knowledge, confidence and complacency of vaccines in the pathways from conspiracy beliefs to vaccine hesitancy. Vaccines (Basel). 2021;9(11):1342. doi: 10.3390/vaccines9111342. 34835273 PMC8624917

[22] Scharff DP, Mathews KJ, Jackson P, et al. More than Tuskegee: understanding mistrust about research participation. J Health Care Poor Underserved. 2010;21(3):879–897. doi: 10.1353/hpu.0.0323. 20693733 PMC4354806

[23] Borno HT, Andemeskel G, Palmer NR. Redefining attribution from patient to health system—how the notion of “mistrust” places blame on black patients. JAMA Oncol. 2021;7(5):780. doi: 10.1001/jamaoncol.2020.8482. 33662096

[24] Fletcher FE, Jiang W, Best AL. Antiracist praxis in public health: a call for ethical reflections. Hastings Cent Rep. 2021;51(2):6–9. doi: 10.1002/hast.1240. PMC9009418 33840102

[25] Allen JD, Fu Q, Shrestha S, et al. Medical mistrust, discrimination, and COVID-19 vaccine behaviors among a national sample U.S. adults. SSM Popul Health. 2022;20:101278. doi: 10.1016/j.ssmph.2022.101278. 36407121 PMC9652159

[26] Bogart LM, Ojikutu BO, Tyagi K, et al. COVID-19 related medical mistrust, health impacts, and potential vaccine hesitancy among black Americans living with HIV. J Acquir Immune Defic Syndr. 2021;86(2):200–207. doi: 10.1097/QAI.0000000000002570. 33196555 PMC7808278

[27] Thompson HS, Manning M, Mitchell J, et al. Factors associated with racial/ethnic group–based medical mistrust and perspectives on COVID-19 vaccine trial participation and vaccine uptake in the US. JAMA Netw Open. 2021;4(5):e2111629. doi: 10.1001/jamanetworkopen.2021.11629. 34042990 PMC8160590

[28] Morgan KM, Maglalang DD, Monnig MA, et al. Medical mistrust, perceived discrimination, and race: a longitudinal analysis of predictors of COVID-19 vaccine hesitancy in US adults. J Racial Ethn Health Disparities. 2023;10(4):1846–1855. doi: 10.1007/s40615-022-01368-6. 35913543 PMC9341411

[29] Charura D, Hill AP, Etherson ME. COVID-19 vaccine hesitancy, medical mistrust, and mattering in ethnically diverse communities. J Racial Ethn Health Disparities. 2023;10(3):1518–1525. doi: 10.1007/s40615-022-01337-z. 35641735 PMC9154033

[30] Tsui J, Martinez B, Shin MB, et al. Understanding medical mistrust and HPV vaccine hesitancy among multiethnic parents in Los Angeles. J Behav Med. 2023;46(1-2):100–115. doi: 10.1007/s10865-022-00283-9. 35107656 PMC8808279

[31] Qualtrics.com. The leading experience management software. 2024. [cited Jan 19]. Available from https://www.qualtrics.com/

[32] Bass SB, Cabey WV, Kelly PJ, et al. Using diffusion of innovation theory to characterize willingness to receive a COVID-19 booster among vaccinated adults. Ann Behav Med. 2023;57(1):S37.

[33] Bass SB, Kelly PJ, Hoadley A, et al. Mapping perceptual differences to understand COVID-19 beliefs in those with vaccine hesitancy. J Health Commun. 2022;27(1):49–61. doi: 10.1080/10810730.2022.2042627. 35199628

[34] Biasio LR, Bonaccorsi G, Lorini C, et al. Assessing COVID-19 vaccine literacy: a preliminary online survey. Hum Vaccin Immunother. 2021;17(5):1304–1312. doi: 10.1080/21645515.2020.1829315. 33118868 PMC8078752

[35] Bruder M, Haffke P, Neave N, et al. Measuring individual differences in generic beliefs in conspiracy theories across cultures: conspiracy mentality questionnaire. Front Psychol. 2013;4:225. doi: 10.3389/fpsyg.2013.00225. 23641227 PMC3639408

[36] LaVeist TA, Isaac LA, Williams KP. Mistrust of health care organizations is associated with underutilization of health services. Health Serv Res. 2009;44(6):2093–2105. doi: 10.1111/j.1475-6773.2009.01017.x. 19732170 PMC2796316

[37] Boston University School of Public Health. Diffusion of innovation theory. [cited 2024 Jan 19]. Available from https://sphweb.bumc.bu.edu/otlt/mph-modules/sb/behavioralchangetheories/behavioralchangetheories4.html

[38] Columbia University Mailman School of Public Health. K-means cluster analysis. 2016. [cited 2024 Jan 8]. Available from https://www.publichealth.columbia.edu/research/population-health-methods/k-means-cluster-analysis

[39] Smail E, Schneider KE, DeLong SM, et al. Health beliefs and preventive behaviors among adults during the early COVID-19 pandemic in the United States: a latent class analysis. Prev Sci. 2021;22(8):1013–1022. doi: 10.1007/s11121-021-01273-0. 34275054 PMC8286044

[40] Bennett NG, Bloom DE, Ferranna M. Factors underlying COVID-19 vaccine and booster hesitancy and refusal, and incentivizing vaccine adoption. PLoS One. 2022;17(9):e0274529. doi: 10.1371/journal.pone.0274529. 36136997 PMC9498968

[41] Paul E, Fancourt D. Predictors of uncertainty and unwillingness to receive the COVID-19 booster vaccine: an observational study of 22,139 fully vaccinated adults in the UK. Lancet Reg Health Eur. 2022;14:100317. doi: 10.1016/j.lanepe.2022.100317. 35132400 PMC8811487

[42] Galanis P, Vraka I, Katsiroumpa A, et al. First COVID-19 booster dose in the general population: a systematic review and meta-analysis of willingness and its predictors. Vaccines (Basel). 2022;10(7):1097. doi: 10.3390/vaccines10071097. 35891260 PMC9323526

[43] Raut A, Samad A, Verma J, et al. Acceptance, hesitancy and refusal towards COVID-19 vaccination. Clin Epidemiol Glob Health. 2023;21:101283. doi: 10.1016/j.cegh.2023.101283. 37033719 PMC10070193

[44] Betsch C, Renkewitz F, Betsch T, et al. The influence of vaccine-critical websites on perceiving vaccination risks. J Health Psychol. 2010;15(3):446–455. doi: 10.1177/1359105309353647. 20348365

[45] Puri N, Coomes EA, Haghbayan H, et al. Social media and vaccine hesitancy: new updates for the era of COVID-19 and globalized infectious diseases. Hum Vaccin Immunother. 2020;16(11):2586–2593. doi: 10.1080/21645515.2020.1780846. 32693678 PMC7733887

[46] Entradas M. In science we trust: I effects of information sources on COVID-19 risk perceptions. Health Commun. 2022;37(14):1715–1723. doi: 10.1080/10410236.2021.1914915. 33941007

[47] Fridman I, Lucas N, Henke D, et al. Association between public knowledge about COVID-19, trust in information sources, and adherence to social distancing: cross-sectional survey. JMIR Public Health Surveill. 2020;6(3):e22060. doi: 10.2196/22060. 32930670 PMC7511226

[48] Nah S, Williamson LD, Kahlor LA, et al. The roles of social media use and medical mistrust in black Americans’ COVID-19 vaccine hesitancy: the RISP model perspective. Health Commun. 2023;39(9):1833–1846. doi: 10.1080/10410236.2023.2244169. 37551159

[49] Nah S, Williamson LD, Kahlor LA, et al. COVID-19 vaccine hesitancy in Cameroon: the role of medical mistrust and social media use. J Health Commun. 2023;28(9):619–632. doi: 10.1080/10810730.2023.2250287. 37622325

[50] Viskupič F, Wiltse DL, Meyer BA. Trust in physicians and trust in government predict COVID‐19 vaccine uptake. Soc Sci Q. 2022;103(3):509–520. doi: 10.1111/ssqu.13147. 35600052 PMC9115527

[51] Rogers E, Singhal A, Quinlan M. Diffusion of innovations: in an integrated approach to communication theory and research. London, United Kingdom: Routledge Taylor and Francis Group; 2014.

[52] Smith RA, Bone C, Visco A, et al. Skeptical health mavens may limit COVID-19 vaccine diffusion: using the innovation diffusion cycle to interpret results of a cross-sectional survey among people who are socially vulnerable. J Health Commun. 2022;27(6):375–381. doi: 10.1080/10810730.2022.2111619. 35983888

[53] Hunter-Mullis K, Macy JT, Greene A, et al. Perceived COVID-19 vaccine attributes associated with early adoption among adults in rural Indiana. Health Educ Res. 2022;37(6):466–475. doi: 10.1093/her/cyac029. 36242555 PMC9619772

[54] Marquez C, Kerkhoff AD, Naso J, et al. A multi-component, community-based strategy to facilitate COVID-19 vaccine uptake among Latinx populations: from theory to practice. PLoS One. 2021;16(9):e0257111. doi: 10.1371/journal.pone.0257111. 34543291 PMC8452046

[55] Siminoff LA, VonNessen-Scanlin S, Wu H, et al. Developing and delivering a comprehensive vaccine COVID-19 program: rapidVax. CommonHealth. 2022;3(1):1–12. doi: 10.15367/ch.v3i1.502.

[56] “Ron ”CR, Sheppard J, Thomas CB. Community pharmacists and communication in the time of COVID-19: applying the health belief model. Res Social Adm Pharm. 2021;17(1):1984–1987. doi: 10.1016/j.sapharm.2020.03.017. 32247680 PMC7118622

